# Supplementary material for: The Effects of Thermal Pasteurisation, Freeze-Drying, and Gamma-Irradiation on the Antibacterial Properties of Donor Human Milk
Source: Foods. 2021 Sep 2;10(9):2077. doi: 10.3390/foods10092077 (PMC8469727; doi:10.3390/foods10092077)
Supplement: Supplementary file 1 [file foods-10-02077-s001.zip › foods-1316175-supplementary.pdf]

Supplementary material

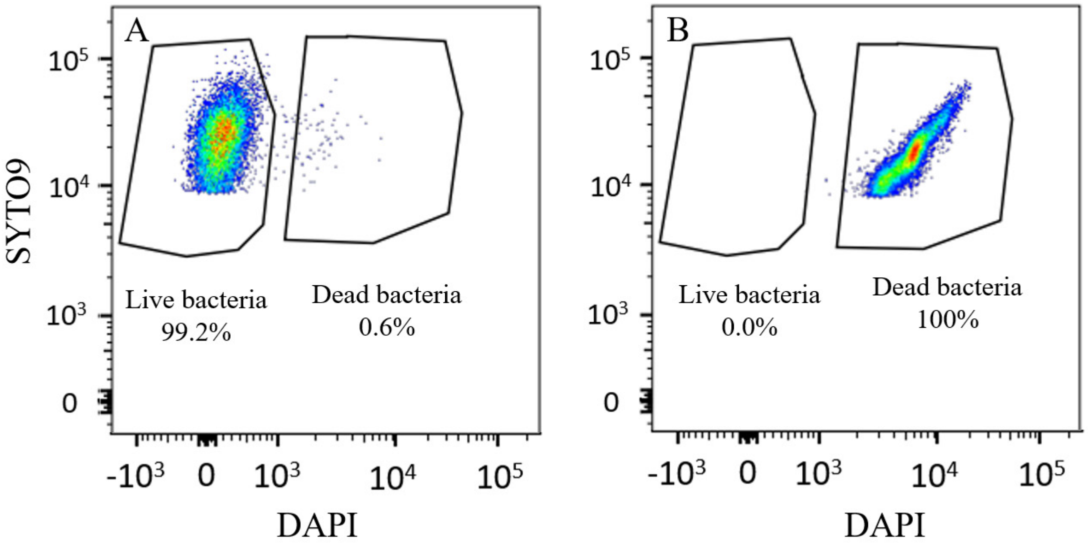

Figure S1: Detection of live and dead bacteria in saline using flow cytometry. Detection of (A) live bacteria in saline and (B) dead bacteria. Bacterial inoculum (10^8 cfu/ml of *S. aureus*) suspended in saline.

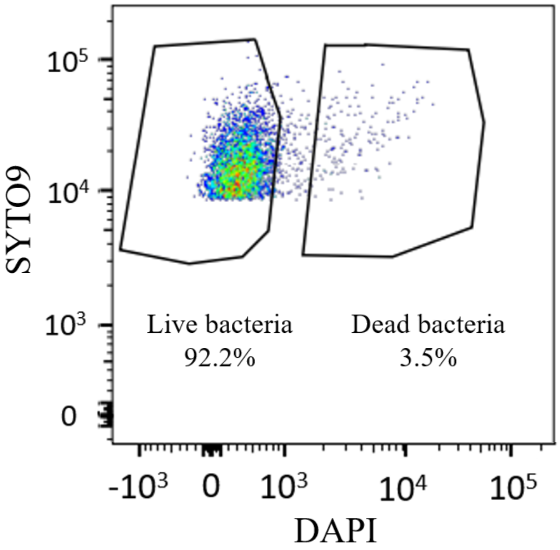

Figure S2: The gating strategy for detection of live/dead bacteria in DHM. Gating strategy uses B530 to detect STYO9 and B585 to gate out the background fluorescence of the milk to improve detection of the bacteria.

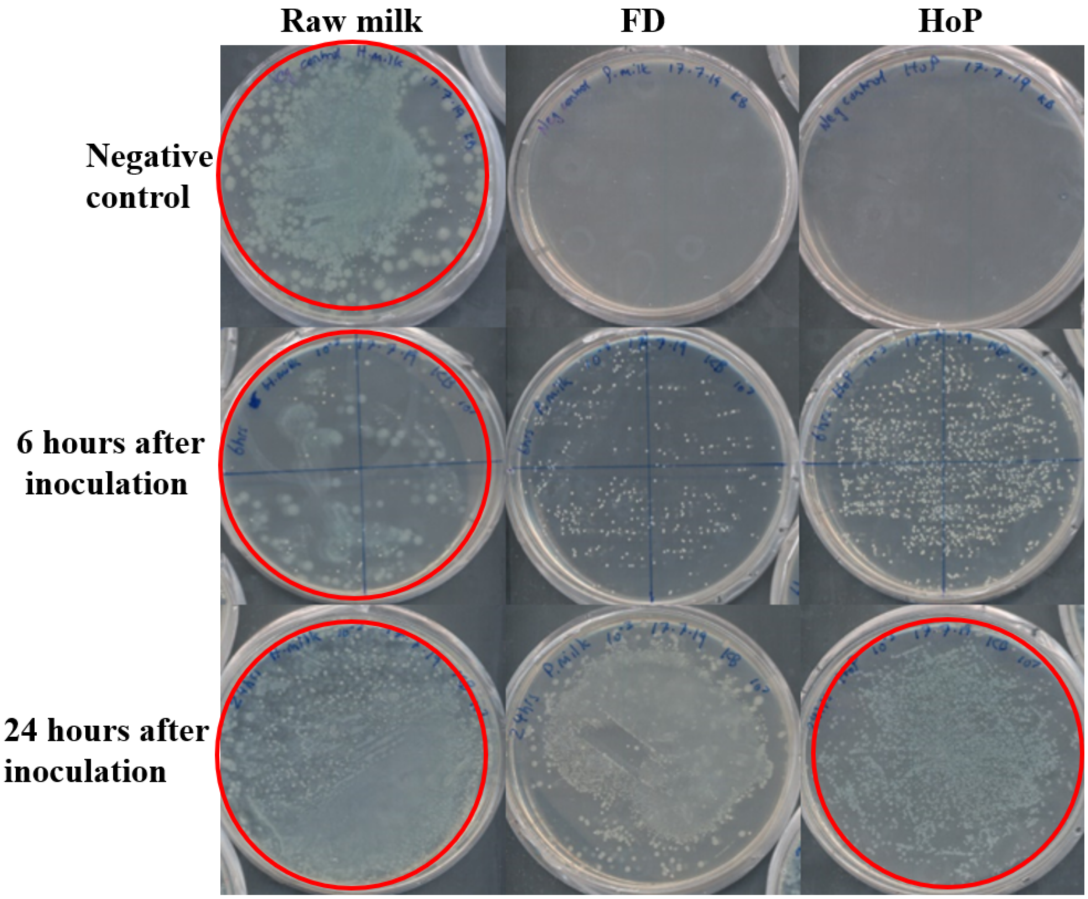

Figure S3: Agar plates showing growth of *S. aureus* in raw DHM and treated DHM. Inoculation quantity of *S. aureus* was 10^7 cfu/ml. Growth of bacteria other than inoculant found in agar plates indicated by red circle.

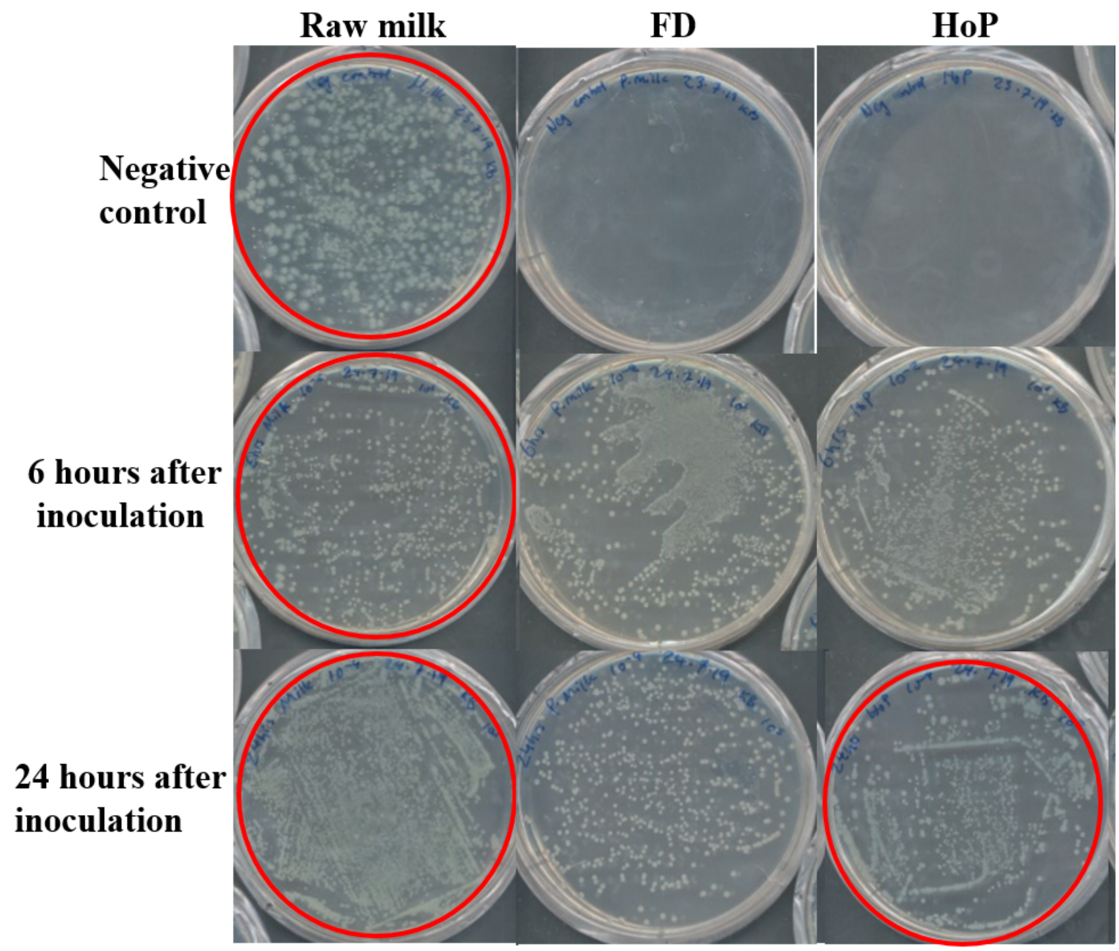

Figure S4: Agar plates showing growth of *E. coli* in raw DHM and treated DHM. Inoculation quantity of *E. coli* was 10^5 cfu/ml. Growth of bacteria other than inoculant found in agar plates indicated by red circle.

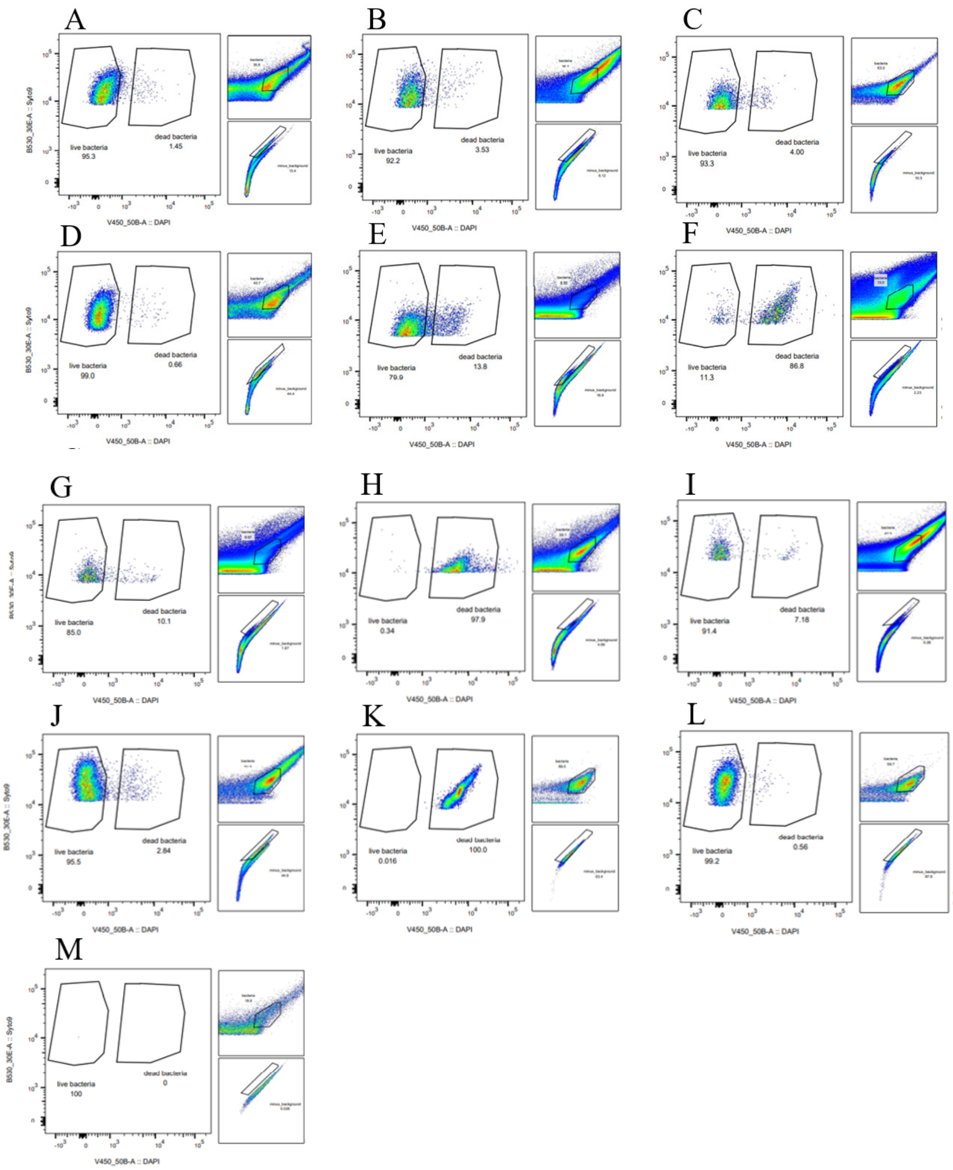

Figure S5: Raw data for flow cytometry for raw and treated DHM. A: Raw DHM, B: Raw DHM, C: HoP DHM, D: HoP DHM, E: FD DHM, F: FD DHM, G: FD+HoP, H: FD+HoP DHM, I: Infant formula J: Infant formula, K: Negative control – LB broth not inoculated, L: Positive control – inoculated LB broth, M: Raw DHM control (not inoculated).
